# 

**Published:** 1855-07

**Authors:** 


### THE OHIO COLLEGE OF DENTAL SURGERY.

**Session of 1855–'56.**

The regular course of lectures in this institution, commences on the first Monday of November, and closes on Tuesday, succeeding the third Monday of February.

**FACULTY.**

JAMES TAYLOR, M. D., D. D. S.
*Professor of the Institutes of Dental Science,*

C. B. CHAPMAN, M. D.,
*Professor of Anatomy and Physiology.*

J. B. SMITH, M. D.,
*Professor of Pathology and Therapeutics.*

JONATHAN TAFT, D. D. S.,
*Professor of Operative Dentistry.*

H. R. SMITH, D. D. S.,
*Professor of Mechanical Dentistry.*

GEORGE WATT, M. D., D. D. S.,
*Professor of Chemistry and Metallurgy.*

The Infirmary will be kept open most of the summer, and during the month of October special attention will be paid to operations before such of the class as can come in by that time. Those desiring *extra* opportunities for practice should avail themselves of this months privileges.

To meet the wants of those engaged in practice, half course tickets are issued, thus enabling them to make out a full course for graduation without leaving their homes and practice for one entire session.

Students should bring with them such instruments as they may have on hand, also the following books, which are more or less used as Text Books: Harris' Principles and Practice of Dental Surgery, Jourdain's Surgical Diseases of the Mouth, Wilson's Anatomy, Carpenter's Physiology, Dunglison's Therapeutics, and Turner's Chemistry.

Candidates for graduation must have attended two full courses of Lectures, the last in this school. Certificates of four years Dental Practice, or of one full course in any respectable Dental or regular Medical College, will be received as equivalent to the first course.

**TERMS OF ADMISSION.**

Tickets are considered as cash, and when not paid for in advance, a note to the Faculty, with approved security, will be required.

| | |
|---|---|
| Tickets for the entire course, and admissions to dissections, | $100 00 |
| Marticulation Ticket, - - - - - - - - | 5 00 |
| Diploma Fee, - - - - - - - | 25 00 |

Good and respectable Board can be had a from $2,50 to $3,00 per week.

JAMES TAYLOR, DEAN.

### PROSPECTUS.

### THE DENTAL REGISTER OF THE WEST

Has now closed its Eighth yearly salutation to the Profession. It was started for a specific object, the diffusion of scientific and practical knowledge.

Those who have regularly read its pages can see how this object has been accomplished. We can only say, that on a retrospect, we feel that our labor has not been in vain; that while we cannot claim all that we desired, yet we see enough to give us cheerful anticipations for the future.

The past eight years have been prolific in Dental improvement and progress, and we feel that our profession now occupies a position far more favorable for advancement.

When the Register was started, eight years since, we had no Western periodical belonging to the Profession and it was with much doubt and hesitation that it would be sustained. It has, however, passed its infancy, and from a small forty-eight page octavo quarterly, it has grown to almost double that size. The wants of the Profession and the amount of readable matter supplied for its pages now demand a still greater enlargement. To meet this want, and supply the profession with such a periodical as is needed in this great valley, we have consented to enlarge still further the Register and issue quarterly a work of one hundred and twelve octavo page at $3·00 per annum.

The Mississippi Valley Association of Dental Surgeons, at their last meeting requested this enlargement and also the increase of price.

The present size (or the size previous to this No.), is as large as can be afforded at $2·00 per annum. If we had a circulation of fifteen hundred or even one thousand paying subscribers we could still enlarge, and as soon as the regular list of subscribers will justify, we shall do so.

The Register will be issued as heretofore, quarterly, on the first of October, January, April, and July. It will be devoted to the interests of Dental Science, and will be made up of original articles; also selected articles from Dental and Medical journals.

The department of Dental Abstracts will still be maintained—using this as a means of condensing that which is most valuable.

We shall as far as possible maintain a department on Dental Chemistry and Metallurgy, and design in each number to appropriate a few pages to a critical review of Dental and Medical publications. This will be particularly applied to all which relates to Dental Surgery. This review we mean shall be impartial, manly and courteous—handling false views and erroneous practice without favor—leaving the truths alone of science to exert their mighty influence in the continued elevation of our Profession.

We hope that eight years labor in connection with the Register, will justify us in saying that our aim is the advancement of Dental Science. If we ever felt that every energy and talent should be thus exerted, we feel it now, and hence, in buckling on our armor for the increased labor assumed, we say to the profession, "Excelsior, Excelsior," and hope that every Dental practitioner will send back the cheerful answer, "Onward, Onward; *we* come to the rescue."

In conclusion, we bespeak, from the profession regular contributions for its pages. Practical, appropriate and concise articles will be thankfully received.

All commuuications should be addressed "Dental Register, Cincinnati, Ohio," or "Dr. James Taylor." The Post Office regulations should be remembered. JAS. TAYLOR.

